# Quality of Life Outcomes and Determining Factors in Breast Cancer Patients Reporting to a Tertiary Care Centre in the Sub-Himalayan Region of India

**DOI:** 10.7759/cureus.55112

**Published:** 2024-02-27

**Authors:** Kaalindi Singh, Kalpana Katoch, Kapil M Pal, Ratti R Negi

**Affiliations:** 1 Radiotherapy, Shri Lal Bahadur Shastri Government Medical College and Hospital, Mandi, IND; 2 Pediatrics, Rohilkhand Medical College, Bareilly, IND

**Keywords:** sub-himalayan, quality of life, breast cancer, br-45, eortc-qlq-c30

## Abstract

Purpose: Breast cancer is the most common malignancy among women worldwide. This study was conducted to determine the quality of life (QOL) outcomes among breast cancer patients in the sub-Himalayan region and, secondly, to identify factors affecting them.

Method: The European Organization for Research and Treatment of Cancer (EORTC) QLQ-C30 and BR-45 questionnaires in English and Hindi translations were used. The BR-45 Hindi translation was obtained using the forward-backward translation method. To check internal consistency and validity, Cronbach’s alpha was employed. EORTC scoring manuals were used to score the questionnaires. The analysis of variance test was used to determine the impact of different treatment and sociodemographic factors on QOL domains.

Results: The English and Hindi translations had Cronbach's alpha values of 0.949 and 0.950, respectively, suggesting that the data gathered were reliable. The mean score for global health status was 64.4 ± 29.7, the functional scale (FS) of QLQ_C30 was 76.9 ± 21.5, the FS of BR45 was 64.6 ± 24.1, the symptom scale (SS) of QLQ_C30 was 20.3 ± 19.2, and the SS of BR45 was 22.5 ± 19.1. Factors adversely affecting global health status included younger age, pre/perimenopausal status, and ongoing chemotherapy. Functional scales were significantly affected by marital status and earlier stages of the disease. Symptom scales were adversely affected by ongoing chemotherapy, an earlier stage of the disease, and a duration of treatment of less than six months.

Conclusion: Tailoring treatment to reduce radiotherapy, surgery, and systemic therapy-related side effects may improve QOL. Counselling and social support groups may help patients cope with the burden of family and societal roles.

## Introduction

Breast cancer is the most common malignancy diagnosed worldwide among women. According to cancer estimates from GLOBOCAN 2020, it has an incidence of 2,262,419 cases annually and a five-year prevalence of 7,790,717 [1]. As per the Indian Council of Medical Research (ICMR) Report 2022, breast cancer was the most common malignancy among Indian females. The estimated number of breast cancer cases among females in the year 2022 was 216,108, the crude incidence rate was 30.4, and the cumulative risk was 1 in 29 [2].

Multimodal treatment with surgery, chemotherapy, radiotherapy, and hormone therapy has improved survival and reduced the local recurrence risk in carcinoma breast [3-6]. With increased survival, quality of life (QOL) post-treatment has become progressively more important [7-9]. Several questionnaires have been designed to assess the QOL among cancer patients. One such tool is the European Organization for Research and Treatment of Cancer (EORTC) module QLQ-C30 [10] and carcinoma breast-specific BR-23 [11]. Both modules have been studied in the Indian population, and despite cultural and linguistic differences, both modules are valid and reliable for testing the QOL among Indian patients [12-14].

However, with advancements in treatment modalities, the tools used to evaluate QOL also need perpetual updation; thus, recently, EORTC introduced the BR-45 module, which provides a more accurate and comprehensive assessment of the impact of current treatment modalities on patients' QOL [15]. It has 23 items from the BR-23 and 22 new items. In this study, the EORTC QLQ-C30 and BR-45 were used to calculate QOL scores of patients, and factors that had a significant impact on the QOL of breast cancer patients were also assessed.

## Materials and methods

This cross-sectional study was conducted among breast cancer patients reporting to a tertiary care cancer centre (TCCC) in the sub-Himalayan region from April 2021 to December 2021.

Measurement tool

The EORTC QLQ-C30 [10], BR-23 [11], and BR-45 [15] questionnaires were used for QOL assessment after obtaining appropriate permission from the EORTC Quality of Life Group via request IDs 71875 and 71865, respectively. The QLQ-C30 [10] is composed of both multi-item scales and single-item measures. These include five functional scales, three symptom scales, a global health status/QOL scale, and six single items. All scales and single-item measures range in score from 0 to 100. A high scale score represents a higher response level. Thus, a high score for a functional scale represents a high/healthy level of functioning, a high score for the global health status/QOL represents a high QOL, but a high score for a symptom scale/item represents a high level of symptomatology/problems. The EORTC QLQ-C30 scoring manual was used for score calculations [16].

The QLQ-BR45 [15] incorporates nine multi-item scales to assess body image, sexual functioning, breast satisfaction, systemic therapy side effects, arm symptoms, breast symptoms, endocrine therapy symptoms, skin mucosis symptoms, and endocrine sexual symptoms. In addition, single items assess sexual enjoyment, future perspective, and being upset by hair loss. The EORTC QLQ-C30 and BR-45 scoring manual [16] were used for score calculation.

Translation method

The English version of the BR-45 [15] was translated into Hindi by two independent translators who were well-versed in both languages. The first translation was regarded as Version 1 of the translation. Version 1 was checked for gross errors or misrepresentations by the study investigators and modified accordingly. It was then given to two individuals who were also well versed in both languages and were unaware of the original English questionnaire for back translation to English. Thus, Versions 2a and 2b of the translation were obtained. Versions 2a and 2b were compared with the original English questionnaire for any gross deviations or changes in the meaning of the questions. All the investigators found them satisfactory with minor changes, and the final Hindi version was administered to 17 patients.

Data collection

Patients who could read and write in either English or Hindi or could understand the questionnaire with some assistance were included in the study and administered the questionnaires in the Outpatient Department of Radiotherapy at a TCCC in a private and confidential setting. Patients who could not understand the questionnaire or were unwilling to participate in the study were excluded. Along with the questionnaire, a pre-designed pro forma comprising sociodemographic and clinical information was also filled out for each patient. The demographic and clinical characteristics of the patients have been summarised in Tables 1, 2.

Statistical analysis

Cronbach's Alpha was utilised to evaluate the reliability and internal consistency of the questionnaire. A questionnaire's value of Cronbach's Alpha higher than 0.70 suggests that the items in the questionnaire are highly connected to one another and accurately assess the construct. After calculating Cronbach's Alpha, additional analysis at the item level was done to locate the items contributing to a lack of internal consistency. Examining the alpha value at the item or statement level, none of the items' internal consistency was low enough for removal. Hence, all 75 statements were considered for analysis.

The QOL scores were calculated using the EORTC scoring manuals for both questionnaires [16]. The impact of different sociodemographic and clinical variables on QOL was evaluated using the analysis of variance (ANOVA) F-test.

## Results

The questionnaires were completed by 47 patients. The mean age was 55.6 years. The demographic and clinical profile of the patients is summarised in Tables 1, 2. 

**Table 1:** Sociodemographic Profile of Patients

Sociodemographic characteristics		Frequency	Percentage
Age	<50 years	18	38.3%
	>50 years	29	61.7%
	Total	47	100.0%
Gender	Male	1	2.1%
	Female	46	97.9%
	Total	47	100.0%
Marital status	Married	38	80.9%
	Unmarried	2	4.3%
	Widowed	7	14.9%
	Total	47	100.0%
Habitat	Rural	40	85.1%
	Urban	7	14.9%
	Total	47	100.0%
Family structure	Joint	32	68.1%
	Nuclear/living alone	15	31.9%
	Total	47	100.0%
Insurance/government scheme	Yes	22	46.8%
	No	25	53.2%
	Total	47	100.0%
Socioeconomic status	Lower	1	2.1%
	Lower middle	15	31.9%
	Upper lower	30	63.8%
	Upper	1	2.1%
	Total	47	100.0%

**Table 2:** Clinical Profile of Patients

Variables		Frequency	Percentage
Stage	Stage I	8	17.0%
	Stage II	22	46.8%
	Stage III	16	34.0%
	Stage IV	1	2.1%
	Total	47	100.0%
Radiotherapy	Yes	39	83.0%
	No	8	17.0%
	Total	47	100.0%
Duration of treatment	<6 months	7	14.9%
	>6 months	40	85.1%
	Total	47	100.0%
Stage of treatment	Follow-up	30	63.8%
	On chemo	17	36.2%
	Total	47	100.0%
Comorbidities	Yes	17	36.2%
	No	30	63.8%
	Total	47	100.0%
Menopausal status	Pre/perimenopausal	7	14.9%
	Post-menopausal	40	85.1%
	Total	47	100.0%

All questionnaires were evaluated; of these, 30 questionnaires were answered in English and 17 in Hindi. Cronbach’s Alpha value for English respondents was 0.949, and for respondents in Hindi, it was 0.950. The difference was statistically non-significant. Since both values were more than 0.70, the data were considered fit for further analysis.

The mean score for global health status was 64.4, with a standard deviation of 29.7. A summary of the final scores calculated per the scoring manual is shown in Table 3.

**Table 3:** Quality of Life Scores of Patients Global Health Status Score: Range 0-100; Functional Scale: Range 0-100; Symptom scales: Range 0-100. QL2_30: Gobal Health Score of QLQ-C30; FS_30: Functional score of QLQ_C30; FS_45: Functional score of BR45; SS_30: Symptom score of QLQ-C30; SS_45: Symptom score of BR45.

Overall quality of life		Mean scores	SD
Global health status	QL2_30	64.4	29.7
Functional scales	FS_30	76.9	21.5
	FS-45	64.6	24.1
Symptom scales	SS_30	20.3	19.2
	SS_45	22.5	19.1

The most distressing symptoms reported were hair loss, fatigue, arm symptoms, and systemic therapy side effects. The last reported symptoms were sexual discomfort, dyspnoea, and diarrhoea. Among functional scales, patients had high scores for sexual enjoyment, cognitive functioning, role functioning, and social functioning; the lowest scores were obtained for breast satisfaction. The functional and symptom scores are summarised in Table 4 and Table 5, respectively. 

**Table 4:** Quality of Life (QOL) Symptom Scale Scores All symptom scale scores range from 0 to 100, with a higher score indicating a high level of symptomatology or problems.

QOL (symptom scale)	Mean scores	SD
Fatigue	34.7	27.9
Nausea and vomiting	10.6	19.8
Pain	30.5	24.6
Dyspnoea	7.1	16.9
Insomnia	24.1	29.2
Appetite loss	19.9	33.1
Constipation	19.9	30.0
Diarrhoea	9.9	21.9
Financial difficulties	26.2	36.7
Systemic therapy side effects	30.9	24.6
Upset by hair loss	39.7	45.9
Arm symptoms	33.8	27.1
Breast symptoms	20.2	21.1
Endocrine therapy symptoms	22.6	16.8
Skin mucosis symptoms	14.2	19.9
Endocrine sexual symptoms	–3.7	23.4

**Table 5:** Quality of Life (QOL) Functional Scale Scores All functional scale scores and single-item measures range from 0 to 100. A high score for functional items suggests a high/healthy level of functioning.

QOL (functional scale)	Mean scores	SD
Physical functioning	76.0	19.8
Role functioning	80.5	27.7
Emotional functioning	66.8	31.6
Cognitive functioning	81.5	23.4
Social functioning	79.8	31.1
Body image	71.5	27.8
Future perspective	53.2	36.6
Sexual functioning	60.3	40.0
Sexual enjoyment	98.6	42.8
Breast satisfaction	45.4	36.9

Among factors affecting the QOL, sociodemographic factors such as younger age, married status, and pre/perimenopausal status had a worse impact on QOL. Among disease and treatment-related factors, significant factors were the stage of disease, stage of treatment, chemotherapy administration, and duration of therapy.

Age over 50 years was associated with a higher global health score (GS-30) (p=0.03). Post-menopausal women also had higher global health scores than pre/perimenopausal women (p=0.036). Ongoing chemotherapy was another significant factor reducing Global health status (p=0.04).

As for marital status, married women had significantly lower scores in the functional domain-45 (FS-45), followed by unmarried and highest in widowed (p=0.01), indicating better functioning QOL among unmarried and widowed women than married women.

Patients with stage I and II disease and ongoing chemotherapy had higher symptom scores in EORTC-30 (SS-30) (p=0.04) and EORTC-45 (SS-45) (p=0.02) compared to stage III and IV patients, interpreted as more symptoms and poorer functioning as compared to higher stage patients. Patients receiving chemotherapy also had significantly lower function scores in EORTC-45 (FS-45) (p=0.02). A prolonged duration of treatment (more than six months) was associated with lower scores in the Symptom score of EORTC-30 (p=0.051), indicating fewer symptoms in patients under treatment for long durations.

Other factors, such as socioeconomic status, presence or absence of insurance, family structure, presence of co-morbidities, and region of residence, i.e., rural or urban, did not significantly impact the QOL.

## Discussion

The significance of QOL among breast cancer patients cannot be overstated, especially with advancements in treatment modalities that have significantly improved survival rates. Based on a report by Montazari et al. [17], it is highly likely that when a woman is diagnosed with breast cancer, other members of her family may also develop some health problems. In India, socio-cultural norms prescribe distinct roles for women in a family compared to their Western counterparts. Additionally, the availability of treatment facilities, economic factors, and the presentation stage vary considerably across different regions in India. It is pertinent to note that the QOL assessment tools developed in Western countries may not directly apply to Indian women due to these differences.

In this study, the majority of women were residing in rural areas (85.1%), most belonged to the upper-lower socioeconomic status as per Kuppuswamy’s classification (63.8%) [18], and only 46.8% of women had health insurance. Most were married (80.9%); unlike in Western countries, a joint family was the most common family structure (68.1%). The majority of patients interviewed were in the early stage (63.8%), all radically treated patients had undergone a modified radical mastectomy, 85% of patients had received radiotherapy during radical treatment, and all had received chemotherapy. Most of the patients were on follow-up at the time of the interview.

The QOL tool administered was the English and Hindi translations of EORTC QLQ-C30 [10] and BR-45 [15] to 30 and 17 patients, respectively. The internal consistency was excellent and comparable between the two versions. Compared to a previous QOL study from South India [19] that used the EORTC QLQ-C30 [10] and BR-23 tool [11], the Global health scores were lower, Functional scores were lower, and Symptom scores were higher, indicating a poorer QOL among women in the region.

Several studies have assessed the impact of various treatment and environment-related factors on the QOL in Indian women. Smitha et al. [20] showed that a prolonged duration of therapy led to a poorer QOL among breast cancer patients: patients interviewed after ≥5 cycles of chemotherapy had lower functional scores for future perspective, physical and role functioning, and higher scores for symptoms of hair loss and arm symptoms, compared to patients interviewed at ≤2 cycles. Corroborating these findings, Parmar et al. [12] reported that global QOL, physical, sexual, and role functioning deteriorated with chemotherapy. In our study, ongoing chemotherapy adversely affected global health status and functional and symptom scores, reinforcing the earlier findings.

The effect of the type of surgery on QOL is still being determined, as different studies have reported different results [12,19,21]. Parmar et al. reported superior body image among women who underwent breast conservation treatment compared to those with mastectomy; however, physical, emotional, and cognitive functions were not related to the type of surgery. Dubashi et al. [19] reported higher global health status and sexual functional and sexual enjoyment scores among women who underwent mastectomy; arm symptoms and pain were higher in the breast conservation group. Patients who underwent breast reconstruction surgery were found to be more satisfied with their QOL in another study [22]. In our study, all patients underwent modified radical mastectomy (MRM), and the effect of different types of surgeries on QOL could not be assessed.

Wadasadawala et al. [23] investigated the effect of reduction in radiation treatment field size by accelerated partial breast irradiation (APBI) on QOL. They found that patients treated with APBI had better social functioning, body image, and financial outcomes than those who received whole breast radiotherapy (WBRT). In this study, all patients who underwent radiotherapy received whole breast radiotherapy.

Other social and environmental factors that have been shown to reduce QOL include unemployed status, financial difficulties, duration of disease, higher grade of tumour [13,20], marital status [19], sociocultural status, age of the patients [24], and ongoing chemotherapy [12,20,23].

In our study, younger age, pre/perimenopausal status, and ongoing chemotherapy treatment harmed the global health score. A similar finding was reported by Sharma et al. [23], where younger patients had uniformly lower scores for global and functional outcomes. A study by Burgess et al. [25], which studied the five-year prevalence of depression and anxiety among early-stage breast cancer patients after treatment, showed that younger patients had a significantly higher predisposition to develop long-term depression. Poorer QOL among pre/perimenopausal women may be understood by the changes in menstrual cycles and the onset of menopausal symptoms due to chemotherapy, which may lead to stress and anxiety, unlike post-menopausal women who have already experienced the perimenopausal symptoms.

Functional scores were worse in married women and women with early-stage disease. Married patients had lower QOL scores, likely due to the increased burden of family commitments and responsibilities and the inability to cope with these due to the disease process, as reported in previous studies [20]. Domains that most affected functional outcomes were breast satisfaction, future perspective, sexual functioning, and emotional functioning. These findings were similar to previous reports [13,21].

Symptom scores were significantly affected by earlier stages of the disease, ongoing chemotherapy, and a duration of treatment of less than six months. The finding of worse QOL in our patients with earlier stages of illness and a course of therapy less than six months was contrary to previous studies. This could be because the majority of the patients were in the early stages, and many of the stage I and II patients interviewed were undergoing chemotherapy at the time of the interview when treatment-related toxicity was more severe. The most common symptoms that reduced patients' QOL were hair loss, fatigue, arm symptoms, and systemic therapy side effects. Other previously reported factors such as financial, occupational, grade of tumour, and socio-cultural factors were not found significant in this study.

A significant limitation of this study was the small sample size. Secondly, the effect of different treatment modalities could not be compared as all patients received similar treatments, i.e., modified radical mastectomy, whole breast radiotherapy, and chemotherapy.

In this era of individualised treatment, tailoring treatment to patient-specific factors may prevent over-treatment and reduce treatment-related toxicity, thus improving the QOL. The use of chemotherapy may be limited by risk stratification and genomic profiling. Similarly, reducing radiotherapy fields or omitting radiotherapy in patients with early-stage disease can also reduce local radiotherapy-related side effects such as arm symptoms and breast symptoms. However, these modifications are limited by the availability and accessibility of diagnostic and treatment modalities, emphasising the need to further strengthen the health system and expertise. Other interventions that can improve the QOL include a strong social support system, family discussions, and counselling regarding the disease, which can help patients cope with their social and familial responsibilities.

## Conclusions

The woman is typically the primary caregiver in a family, so her health is paramount to the overall well-being of other family members and society. Improvements in the detection rates of breast cancer in early stages, tailored treatment protocols, and a dependable social support system can contribute towards improving the quality of life among these patients.

## References

[1] (2022). GLOBOCAN 2020: estimated cancer incidence, mortality and prevalence worldwide in 2020. GLOBOCAN.

[2] Sathishkumar K, Chaturvedi M, Das P, Stephen S, Mathur P (2022). Cancer incidence estimates for 2022 &amp; projection for 2025: result from National Cancer Registry Programme, India. Indian J Med Res.

[3] Winchester DP, Cox JD (1998). Standards for diagnosis and management of invasive breast carcinoma. American College of Radiology. American College of Surgeons. College of American Pathologists. Society of Surgical Oncology. CA Cancer J Clin.

[4] Early Breast Cancer Trialists’ Collaborative Group (2002). Multi-agent chemotherapy for early breast cancer. Cochrane Database Syst Rev.

[5] Henderson IC (1994). Adjuvant systemic therapy for early breast cancer. Cancer.

[6] Overgaard M, Hansen PS, Overgaard J (1997). Postoperative radiotherapy in high-risk premenopausal women with breast cancer who receive adjuvant chemotherapy. Danish Breast Cancer Cooperative Group 82b Trial. N Engl J Med.

[7] Whelan TJ, Levine M, Julian J, Kirkbride P, Skingley P (2000). The effects of radiation therapy on quality of life of women with breast carcinoma: results of a randomized trial. Ontario Clinical Oncology Group. Cancer.

[8] Stefanek ME, Derogatis LP, Shaw A (1987). Psychological distress among oncology outpatients Psychosomatics.

[9] Zabora JR, Smith ED (1997). Prevalence of psychosocial distress by cancer site. Proc Am Soc Clin Oncol.

[10] Aaronson NK, Ahmedzai S, Bergman B (1993). The European Organization for Research and Treatment of Cancer QLQ-C30: a quality-of-life instrument for use in international clinical trials in oncology. J Natl Cancer Inst.

[11] Sprangers MA, Groenvold M, Arraras JI (1996). The European Organization for Research and Treatment of Cancer breast cancer-specific quality-of-life questionnaire module: first results from a three-country field study. J Clin Oncol.

[12] Parmar V, Badwe RA, Hawaldar R, Rayabhattanavar S, Varghese A, Sharma R, Mittra I (2005). Validation of EORTC quality-of-life questionnaire in Indian women with operable breast cancer. Natl Med J India.

[13] Deshpande PR, Sheriff MK, Nazir A, Bommareddy S, Tumkur A, Naik AN (2013). Patient-reported quality of life outcomes in Indian breast cancer patients: importance, review of the researches, determinants and future directions. J Cancer Res Ther.

[14] Kannan K, Prashant K, Jogdand Gopal Rao S (2011). Quality of life of women with breast cancer at a tertiary care hospital. Int J Biol Med Res.

[15] Bjelic-Radisic V, Cardoso F, Cameron D (2020). An international update of the EORTC questionnaire for assessing quality of life in breast cancer patients: EORTC QLQ-BR45. Ann Oncol.

[16] Fayers PM, Aaronson NK, Bjordal K, Groenvold M, Curran D, Bottomley A (2001). The EORTC QLQ-C30 Scoring Manual. Published by: European Organisation for Research and Treatment of Cancer, Brussels.

[17] Montazeri A (2008). Health-related quality of life in breast cancer patients: a bibliographic review of the literature from 1974 to 2007. J Exp Clin Cancer Res.

[18] Bairwa M, Rajput M, Sachdeva S (2013). Modified Kuppuswamy’s socioeconomic scale: social researcher should include updated income criteria, 2012. Indian J Community Med.

[19] Dubashi B, Vidhubala E, Cyriac S, Sagar TG (2010). Quality of life among younger women with breast cancer: study from a tertiary cancer institute in south India. Indian J Cancer.

[20] Damodar G, Smitha T, Gopinath S, Vijaykumar S, Rao YA (2013). Assessment of quality of life in breast cancer patients at a tertiary care hospital. Arch Pharm Pract.

[21] Munshi A, Dutta D, Kakkar S (2010). Comparison of early quality of life in patients treated with radiotherapy following mastectomy or breast conservation therapy: a prospective study. Radiother Oncol.

[22] Agarwal P, Patel A K, Saxena A, Mishra A (2011). Assessment of quality of life after breast reconstructive surgery following mastectomy for carcinoma breast. J Surgery Pakistan.

[23] Wadasadawala T, Budrukkar A, Chopra S (2009). Quality of life after accelerated partial breast irradiation in early breast cancer: matched pair analysis with protracted whole breast radiotherapy. Clin Oncol (R Coll Radiol).

[24] Sharma N, Purkayastha A (2017). Factors affecting quality of life in breast cancer patients: a descriptive and cross-sectional study with review of literature. J Midlife Health.

[25] Burgess C, Cornelius V, Love S, Graham J, Richards M, Ramirez A (2005). Depression and anxiety in women with early breast cancer: five year observational cohort study. BMJ.

